# Coupled Hygro-Mechanical Finite Element Method on Determination of the Interlaminar Shear Modulus of Glass Fiber-Reinforced Polymer Laminates in Bridge Decks under Hygrothermal Aging Effects

**DOI:** 10.3390/polym10080845

**Published:** 2018-08-01

**Authors:** Xu Jiang, Chengwei Luo, Xuhong Qiang, Qilin Zhang, Henk Kolstein, Frans Bijlaard

**Affiliations:** 1 Department of Bridge Engineering, College of Civil Engineering, Tongji University, Shanghai 200092, China; jiangxu@tongji.edu.cn (X.J.); jerrycwluo@163.com (C.L.); 2 Department of Structural Engineering, College of Civil Engineering, Tongji University, Shanghai 200092, China; qilinzhang0@vip.sina.com; 3 Faculty of Civil Engineering and Geosciences, Delft University of Technology, 2628CN Delft, The Netherlands; M.H.Kolstein@tudelft.nl (H.K.); F.S.K.Bijlaard@tudelft.nl (F.B.)

**Keywords:** fiber-reinforced polymer composite, interlaminar shear modulus, hygrothermal aging effect, mechanical degradation, short-beam test, finite element method

## Abstract

To investigate the mechanical degradation of the shear properties of glass fiber-reinforced polymer (GFRP) laminates in bridge decks under hygrothermal aging effects, short-beam shear tests were performed following the ASTM test standard (ASTM D790-10A). Based on the coupled hygro-mechanical finite element (FE) analysis method, an inverse parameter identification approach based on short-beam shear tests was developed and then employed to determine the environment-dependent interlaminar shear modulus of GFRP laminates. Subsequently, the shear strength and modulus of dry (0% *M_t_*/*M_∞_*), moisture unsaturated (30% *M_t_*/*M_∞_* and 50% *M_t_*/*M_∞_*), and moisture saturated (100% *M_t_*/*M_∞_*) specimens at test temperatures of both 20 °C and 40 °C were compared. One cycle of the moisture absorption–desorption process was also investigated to address how the moisture-induced residual damage degrades the shear properties of GFRP laminates. The results revealed that the shear strength and modulus of moisture-saturated GFRP laminates decreased significantly, and the elevated testing temperature (40 °C) aggravated moisture-induced mechanical degradation. Moreover, an unrecoverable loss of shear properties for the GFRP laminates enduring one cycle of the moisture absorption–desorption process was evident.

## 1. Introduction

Various types of fiber-reinforced polymer (FRP) composites are being used in different fields of application, ranging from sporting goods to structural materials for the automotive, maritime, and aerospace industries. During the past two decades, in the civil engineering field, FRP bridge decks are increasingly being used for the rehabilitation of old concrete–steel composite bridges and the new construction of pedestrian and highway bridges [1,2,3,4,5,6], due to their various advantages, including [7,8]: a high strength-to-weight ratio, good corrosion resistance, controllable quality, low maintenance cost, and rapid installation with minimum traffic disruption. Although FRP decks are increasingly being used in civil infrastructure applications, their durability and long-term performance are still not comprehensively understood. In such applications, FRP composites are usually exposed to harsh and variable environments with various temperature and moisture ranges (including elevated temperature immersion and ‘‘hot/wet’’ environment exposures). The “hot/wet” environment exposure is supposed to be the severest environmental condition to degrade the mechanical performance of polymeric materials [9,10,11,12,13,14,15,16,17,18,19], which will consequently deteriorate the long-term performance of FRP composite bridges. A comprehensive understanding of the mechanisms of the hygrothermal aging-related degradation on FRP composite materials is necessary for the purposes of evaluating and predicting the service life and durability of FRP infrastructures. In the literature, the influence of moisture absorption on the mechanical properties of FRP composites is well documented [9,10,11,12,13,14,17,20,21,22,23,24,25,26], regarding the tensile, interlaminar shear, and flexural properties as well as toughness. Generally, the combination of moisture and temperature effects seriously degraded the mechanical properties of FRP composites. Due to this mechanism, overall reductions in the modulus, strength, and glass transition temperature of FRP materials were recorded, which were attributed to the plasticizing effect of water absorbed in the matrix. However, results and conclusions vary with the types of matrix (even fibers), fabrication methods, specimen geometries, curing processes, and service environmental conditions.

In order to keep pace with the application of FRP composites in the civil engineering field, this research was undertaken to reveal more knowledge about the environmental degradation of glass fiber-reinforced polymer (GFRP) composite bridges in hot/wet environments. For the use of GFRP bridge decks, GFRP laminates are mainly loaded by wheels in the through-thickness direction. Therefore, the interlaminar shear properties of GFRP materials are of great importance. Currently, standard test methods exist mostly for determination of the in-plane normal and shear modulus, and the strength parameters of FRP composite materials [27]. However, the test method to directly obtain the interlaminar shear modulus is very limited. Failure always occurred through a combination of shear and transverse tension, indicating that a pure shear failure mode was not evident in the test. Therefore, it is imperative that robust methodologies for determining the interlaminar material properties of FRP materials need to be developed. To achieve this objective, in this research, a coupled hygro-mechanical finite element (FE) modeling method was developed. An inverse parameter identification approach to determine the interlaminar shear modulus G13 (G23) of FRP laminates was established.

Through investigation of the sensitivity of the FE analysis [28], it is concluded that the short-beam three-point bending test (rather than the standard Iosipescu test and off-axis tensile test) is sensitive to changes in the interlaminar shear modulus, but relatively insensitive to changes in the other unknown material properties. Hence, the short-beam three-point bending test is the most suitable method to study the interlaminar shear modulus. Thus, short-beam three-point bending tests were conducted in this research to provide the data base to develop the coupled hygro-mechanical finite element model for numerically determining the interlaminar shear modulus of GFRP laminates, and furthermore to systematically study the influence of moisture and temperature effects on the shear modulus and strength of GFRP laminates.

## 2. Experiment

### 2.1. Material

**Figure 1:**
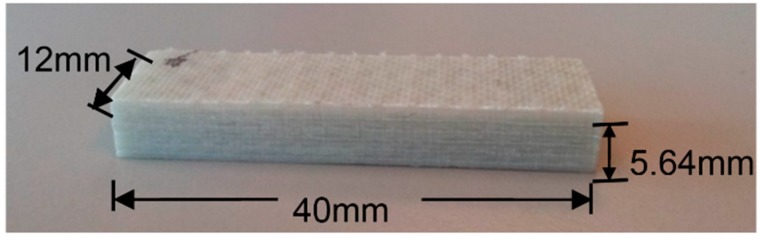
Fiber-reinforced polymer (FRP) laminate short beam specimen.

As aforementioned, to obtain the test database of the coupled hygro-mechanical finite element model and investigate the interlaminar shear property of FRP laminates, three-point bending tests of short-beam GFRP specimens are firstly conducted. The whole test procedure follows the test standard ASTM D2344/D2344M-00 [29]. The GFRP laminates studied in this paper were manufactured by resin vacuum infusion (Infra Composite BV, Breukelen, The Netherlands) using polyester, and then cut into specific dimensions (see Figure 1). The Tg (glass transition temperature) of GFRP is 78 °C, and the maximum working temperature could be 63 °C, which is 15 °C lower than Tg.

The 5.64-mm thick specimen is composed of six layers of standard 0.94 mm EQX1200. The layup configuration of each piece of the standard 0.94-mm EQX1200 is illustrated in Table 1, which is a glass fiber-reinforced polymer composite (54% glass content by weight). The mechanical properties of the GFRP laminates supplied by the manufacturer are shown in Table 2. As shown in Table 1, the nominal length and width of the specimens are selected to be 12 mm and 40 mm.

### 2.2. Methodology

**Figure 2:**
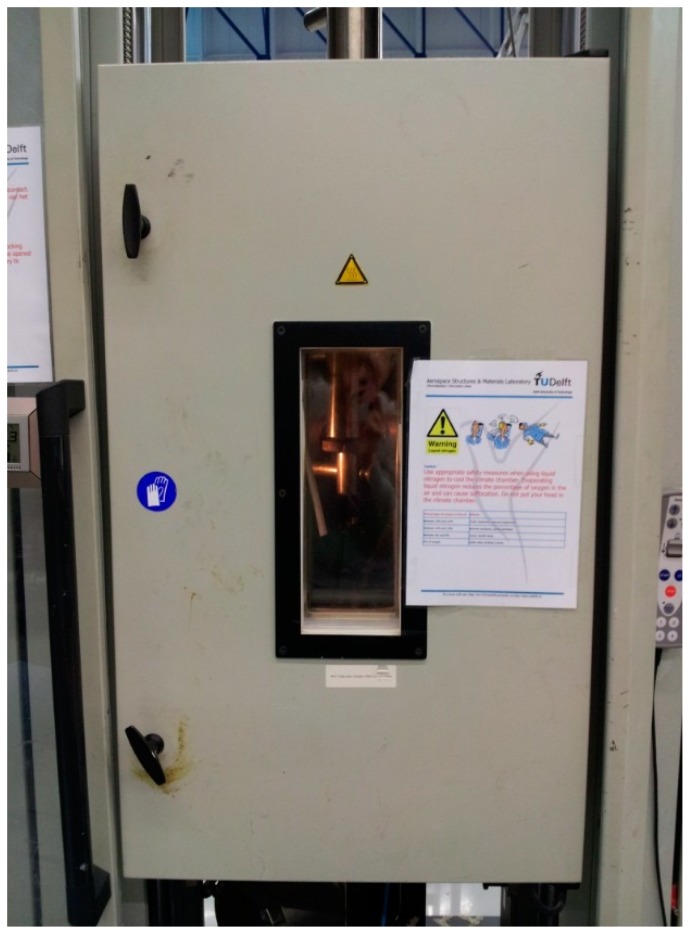
Climate chamber during short-beam tests.

The numbering of specimens with regard to the moisture uptake content, test temperature, absorption/desorption process, and replicated number is listed in Table 3. Two test temperatures, 20 °C and 40 °C, are proposed for the short-beam three-point bending tests, which are controlled by a climate chamber with the tolerance of ±2 °C, as shown in Figure 2. During the short-beam tests, a temperature sensor is attached to the tested GFRP specimen. The test is conducted when the temperature of the specimen reaches the proposed value.

As listed in Table 3, the test under each condition is repeated five times to investigate the deviation of test results. The hygrothermal aging condition (40 °C-water) is supposed to be a relatively severe hot/wet condition for GFRP laminates, as stated in previous research [30]. In total, 70 pieces of specimens are prepared. During the hygrothermal aging procedure, all of the specimens are immersed in water at a temperature of 40 °C, except for the S-0-20 °C and S-0-40 °C specimens, which are the as-received reference specimens (Set-1 in Table 3). The as-received specimens are stored in the laboratory environment. As measured after the moisture desorption, the moisture contents of the specimens are approximately zero. For the other specimens (as illustrated in Table 3), the Set-2 specimens (S-30%-20 °C and S-30%-40 °C) are tested at the 30% relative moisture uptake content. The Set-3 specimens (S-50%-20 °C and S-50%-40 °C) are tested at the 50% relative moisture uptake content. The Set-4 specimens (S-100%-20 °C and S-100%-40 °C) are tested at the moisture saturation level (100% relative moisture uptake content), and the above test series is considered as the moisture absorption process. Then, the rest specimens are all taken out of the hygrothermal aging environment, and put into an oven at a temperature of 42 °C to be dried. This is considered the moisture desorption process. In this way, the Set-5 specimens (S-50%-20 °C-D and S-50%-40 °C-D) are tested at 50% relative moisture uptake content after a certain time of moisture desorption. Subsequently, the Set-6 specimens (S-30%-20 °C-D and S-30%-40 °C-D) are tested at 30% relative moisture uptake content after the moisture desorption. The Set-7 specimens (S-0-20 °C-D and S-0-40 °C-D) are the fully dry specimens after one cycle of the moisture absorption–desorption process. Herein, the symbol “D” indicates moisture desorption. During the whole aging procedure, the moisture uptake content of each specimen is recorded by using the gravimetric test method [30]. The moisture uptake content (*Mt*) of each specimen is calculated according to its weight before exposure (*w_0_*) and after exposure (*w_t_*) as follows:

(1) $$M_{t}=100\times \left(\frac{w_{t}-w_{0}}{w_{0}}\right)\;$$

**Figure 3:**
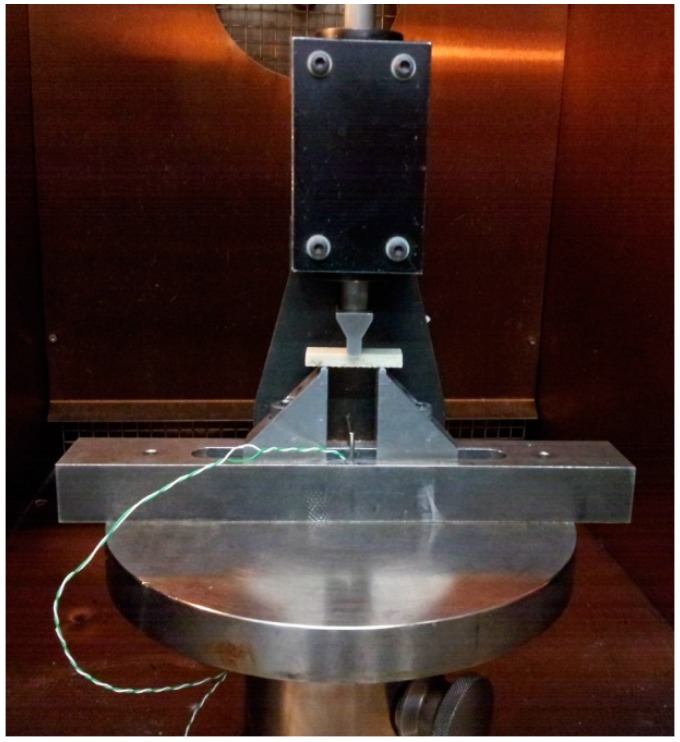
Short-beam shear test device.

The short-beam shear test device is shown in Figure 3.

**Figure 4:**
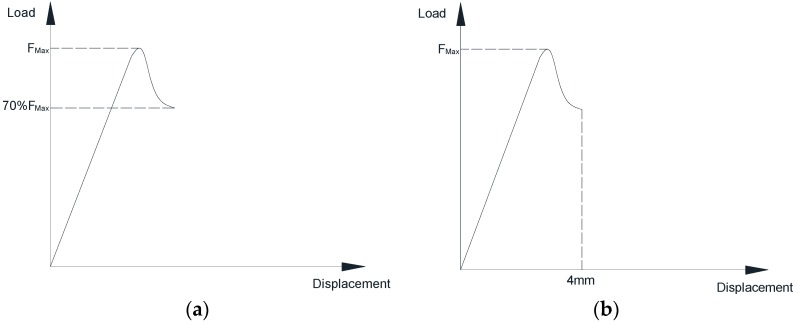
Termination rule for the short-beam shear test. (**a**) Drop to 30% of the maximum load; (**b**) Maximum displacement of 4 mm.

According to ASTM D2344/D2344M-00 [29], the loading span length-to-specimen thickness ratio is four. Consequently, the support span is proposed to be 22.6 mm. It varies among different groups of specimens, since the value of the support span is exactly calculated based on the average thickness of each specimen group. The diameter of the loading nose and supports are 6.00 mm and 3.00 mm, respectively. The crosshead movement speed of testing is set at a rate of 1.0 mm/min. The specimen is deflected until the load drops to 30% of the maximum load or until a maximum displacement of mid-span reaches 4 mm (see Figure 4). The experimental data is recorded per second.

According to the test standard ASTM D2344/D2344M-00 [29], the short-beam shear strength of the FRP laminates can be calculated as follows:

(2) $$F^{sbs}=0.75\times \frac{P_{m}}{b\times h}\;$$

where:

*F^sbs^* = short-beam strength, MPa,
*P_m_* = maximum load observed during the test, N,
*b* = measured specimen width, mm,
*h* = measured specimen thickness, mm.

## 3. Experimental Results and Discussion

**Figure 5:**
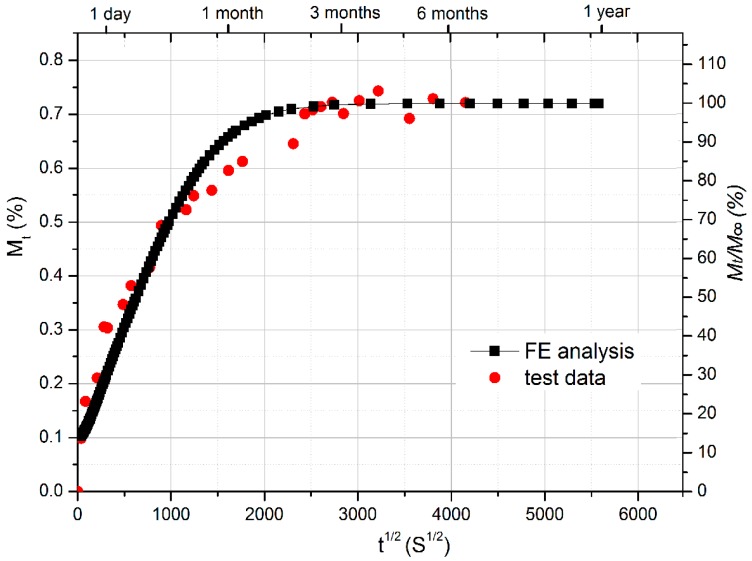
Comparison of moisture uptake curve between test results and finite element (FE) analysis on FRP specimens for short-beam shear tests.

Figure 5 shows the moisture absorption process (red points) of GFRP short-beam specimens immersed in water of 40 °C. Moisture content (*Mt*) is drawn as the function of square root of time. It can be found that the moisture saturation level is about 0.72%.

**Figure 6:**
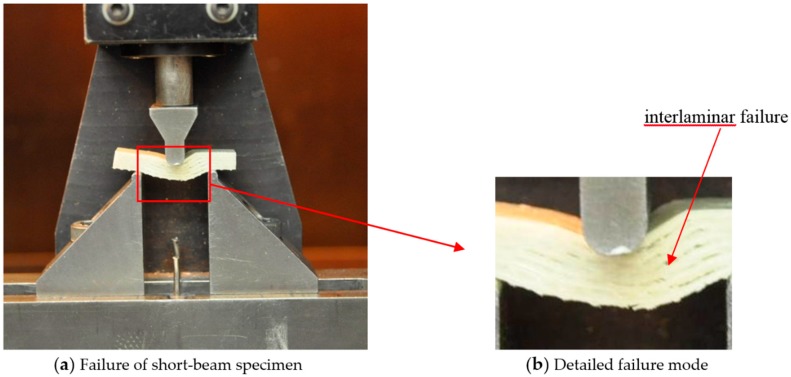
Failure mode of the short-beam shear test specimen.

The typical failure mode of short-beam shear specimens is shown in Figure 6, which is the interlaminar failure through the thickness of GFRP laminates.

**Figure 7:**
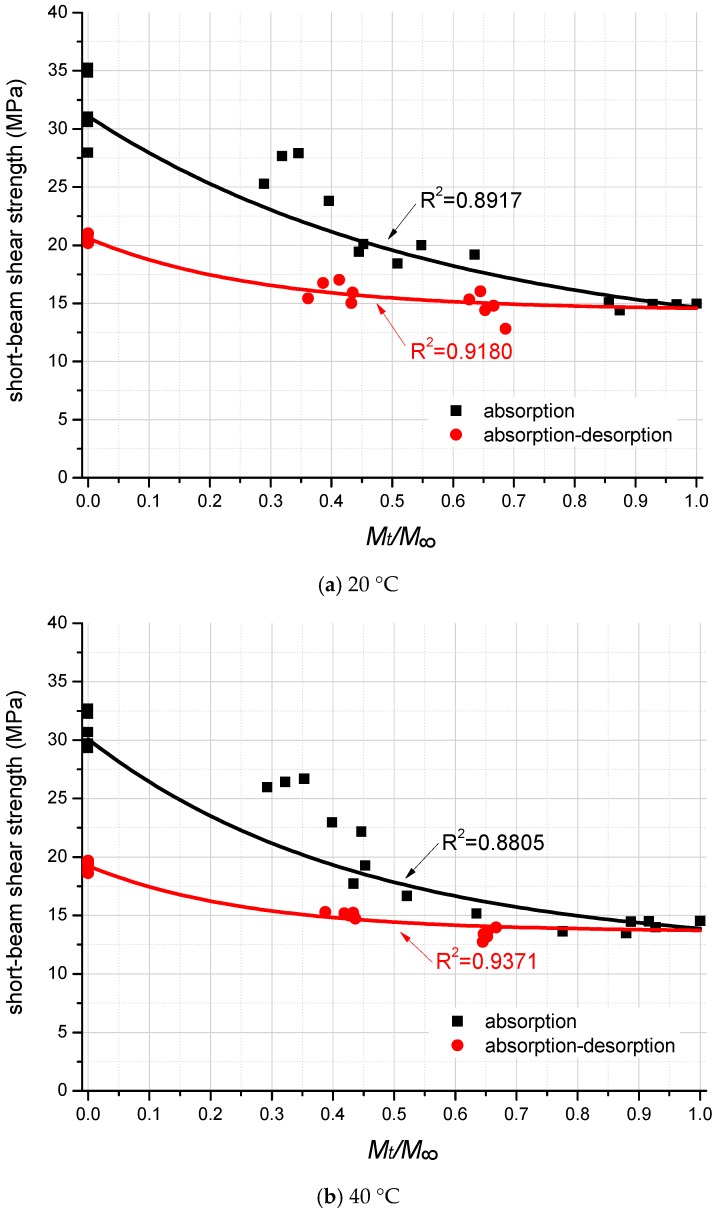
Degradation on the short-beam shear strength of FRP laminates.

Figure 7 shows the mechanical degradation on short-beam shear strength of FRP laminates as a function of moisture uptake content at the test temperatures of 20 °C and 40 °C, respectively.

Predictive equations for the short-beam shear strength degradation as the function of moisture content is curve-fitted by the exponential function using the least square method. They are as follows:

20 °C, absorption process:
  (3) $$S=5.5^{-\left(\frac{M_{t}}{M_{\infty }}-1.76\right)}+11\;$$
20 °C, absorption–desorption process:
  (4) $$S=34^{-\left(\frac{M_{t}}{M_{\infty }}-0.517\right)}+14.4\;$$
40 °C, absorption process:
  (5) $$S=9.6^{-\left(\frac{M_{t}}{M_{\infty }}-1.28\right)}+12\;$$
40 °C, absorption–desorption process:
  (6) $$S=46.8^{-\left(\frac{M_{t}}{M_{\infty }}-0.45\right)}+13.6\;$$

All of the predictive curves are illustrated in Figure 7 for comparison with the experimental results. The R-square values of each curve are also present in Figure 7, which indicates the accuracy of curve fitting on test data points.

As shown in Figure 7a, in the moisture absorption process, the short-beam shear strength is quasi-linearly decreasing from the fully dry specimens to the specimens with about 75% moisture uptake content of the saturated level. Then, test data points distribute stably until reaching the moisture saturated condition (100% *M_∞_*). As listed in Table 4, the short-beam shear strength of the moisture saturated specimens is 15 MPa, which is 53.1% lower than that of the fully dry specimens (32 MPa).

Furthermore, in the moisture absorption process, the test data points are distributed more dispersively, since the moisture uptake process deviates significantly for small scale short-beam specimens. It can be due to the reason that, under the same water aging time, the moisture uptake contents of the individual specimens differ from each other within a certain range. The extent of mechanical degradation is closely related to the moisture content of FRP specimens, but is not related to the aging time.

As for the moisture desorption process, from the saturated condition to the fully dry condition, the short-beam shear strength is slightly increasing, and ends at 21 MPa. It is 34.4% lower than that of the unconditioned dry specimens. This means that one cycle of the moisture absorption–desorption process deteriorated the shear strength of FRP laminates by 34.4% permanently.

Figure 7b presents the same tendency regarding the degradation of the short-beam shear strength of GFRP laminates at 40 °C. As listed in Table 4, the higher temperature (40 °C) only slightly deteriorates the shear strength of GFRP specimens, which implies that the influence of temperature is not as significant as the influence of moisture.

## 4. Coupled Hygro-Mechanical FE Method on Determination of the Interlaminar Shear Modulus of FRP Laminates

For the coupled hygro-mechanical FE method, it is realized in the following steps. The first step is modeling moisture transport through FRP structures in order to determine the moisture concentration distribution across the cross-sections as a function of time. The material parameters required for the transient diffusion FE analysis are moisture diffusion coefficients and solubility, which can be obtained from short-term gravimetric experiments, as stated in previous research [30]. From the moisture diffusion analysis, the moisture concentration distribution across the FRP section can be read into the stress analysis as a predefined field variable. Then, the environment-dependent mechanical behavior of FRP structures can be investigated using the FE stress–stain analysis based on this predefined field. The input moisture-dependent material properties of FRP composites are obtained by material tests (such as flexural test, tensile test, and short-beam shear test).

**Figure 8:**
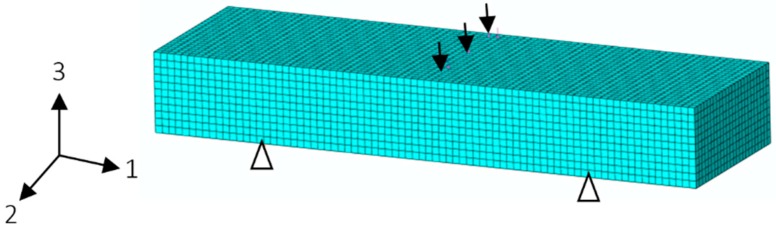
FE model of the short-beam specimen.

In this research, the FE modeling is conducted by employing the FE software ABAQUS. Firstly, the moisture diffusion process of the short-beam GFRP specimen (FE model is shown in Figure 8) is simulated using the transient-field FE diffusion analysis. The type of finite elements is C3D8R. The GFRP material is modeled as an orthotropic material. Moisture diffusion coefficients in three directions are input as *D*_1_ = 9.607 × 10^−6^ mm^2^/s, *D*_2_ = 9.631 × 10^−6^ mm^2^/s, and *D*_3_ = 0.318 × 10^−6^ mm^2^/s (40 °C-water aging condition), which are obtained from the previous research [30].

From Figure 5, good agreement is evident between the experimental results and FE simulation. From the transient-field FE moisture diffusion analysis, the moisture concentration distribution across the short-beam GFRP specimen section is obtained as a function of time, which can be read into the following stress–strain analysis as a predefined field variable at different time intervals. To determine the environment-dependent interlaminar shear modulus of FRP laminates, the coupled hygro-mechanical FE modeling method is employed herein, which was already well developed and validated by flexural tests in the previous research [31]. For instance, at the test temperature of 20 °C and during the moisture absorption process, the flexural modulus (E11 and E22) of GFRP laminates with a nominal moisture content (*M*_t_/*M*_∞_) from 0% to 100% can be calculated using the predictive equations (available in the Ref. [31] of Jiang et al.). It is employed as field-dependent input values for material properties of the FE model herein, which means the flexural modulus of each element is determined by the local moisture concentration. Other material properties are determined according to Table 5, which is supplied by the manufacturer. Depending on the sensitivity analysis, determination of the interlaminar shear modulus is not sensitive to the variation of these material properties. Consequently, the interlaminar shear modulus of GFRP laminates is determined by fitting the coupled hygro-mechanical FE analysis results to the short-beam shear test data.

**Figure 9:**
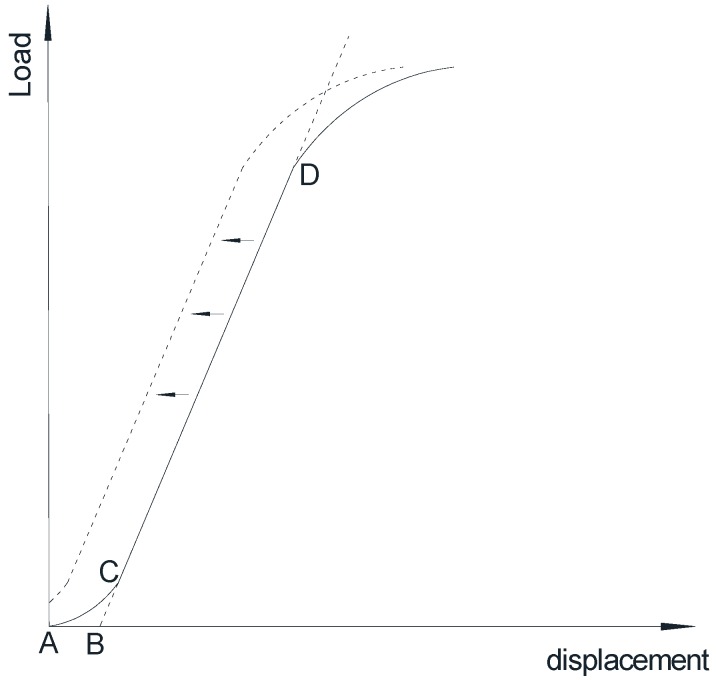
Offset of the experimental load-displacement curve.

According to the test standard ASTM D790-10 [32], as illustrated in Figure 9, the initial non-linear stage of test results is an artifact caused by a take-up of slackness and realignment of the specimens, which does not represent the properties of FRP material. In order to obtain correct values for the material properties, this curve must be offset to the corrected zero point (point B in Figure 9). For each test, the initial non-linear regions are different from each other. To make easy comparisons, all of the experimental curves are offset from B to A, to make the extension line of the linear CD region exactly through the zero point of the coordinates.

**Figure 10:**
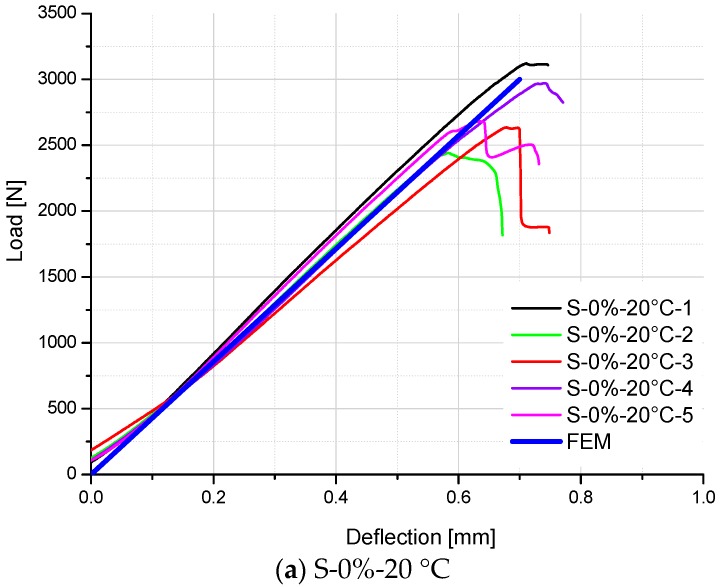

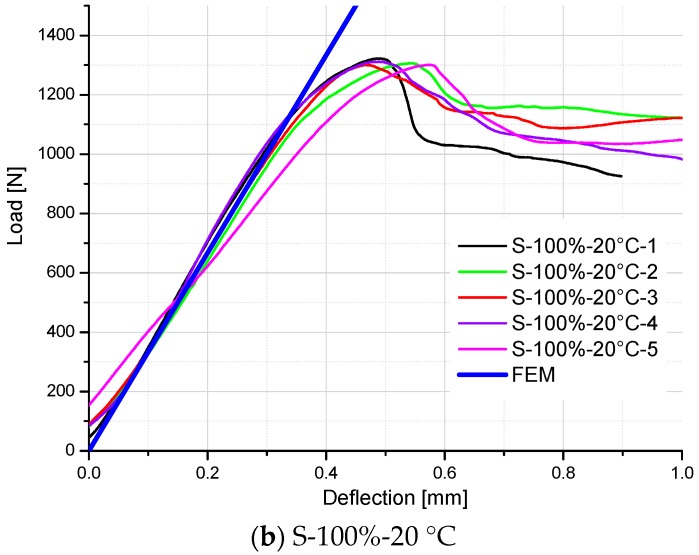
Comparison of load–deflection curves between FE analysis and test results of S-0%-20 °C specimens and S-100%-20 °C specimens.

Furthermore, it is assumed that degradation of interlaminar shear modulus follows a linear relationship with nominal moisture content, which has the same tendency as that obtained from the flexural modulus of GFRP laminates [31]. Subsequently, the interlaminar shear modulus of GFRP specimens with 0% moisture content (S-0%-20 °C) is firstly determined by fitting the FE load–deflection curve to test results, as shown in Figure 10a. Accordingly, the shear modulus G13 (G23) is numerically determined as 1200 MPa. In the same way, the shear modulus G13 (G23) of GFRP specimens with the 100% moisture content (S-100%-20 °C) is determined as 800 MPa (Figure 10b).

The predictive equation for the interlaminar shear modulus of FRP laminates at the test temperature of 20 °C and during the moisture absorption process can be gained as follows:

(7) $$G_{23}=G_{13}=-\frac{400\times M_{t}}{M_{\infty }}+1200\;$$

**Figure 11:**
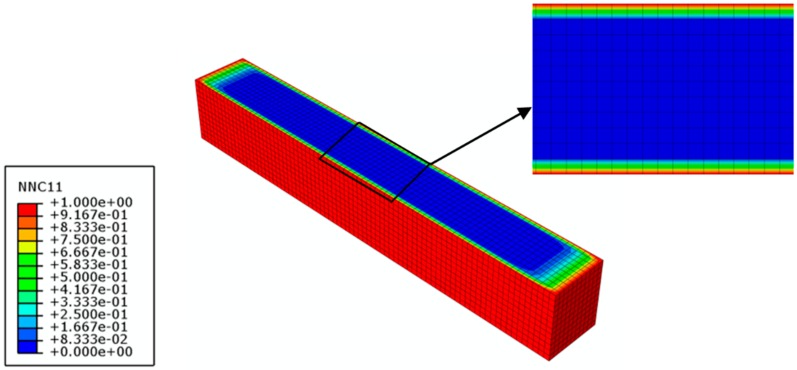
Moisture concentration distribution across the mid-plane of the GFRP specimen with 30% moisture uptake content (time = 26 h).

**Figure 12:**
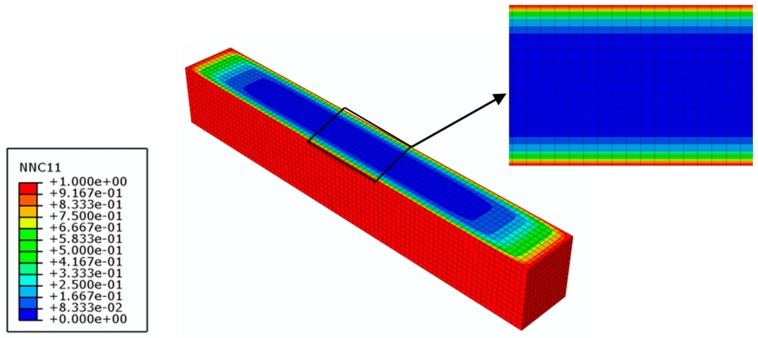
Moisture concentration distribution across the mid-plane of the GFRP specimen with 50% moisture uptake content (time = 107 h).

To validate Equation (7), the other two exposure time intervals (30% *M_t_*/*M_∞_* and 50% *M_t_*/*M_∞_*) are employed. As aforementioned, the moisture diffusion process of the FRP specimen is firstly modeled by the transient-field FE diffusion analysis. According to the moisture diffusion analysis, the moisture concentration distributions across the mid-plane of the FRP specimens are presented in Figure 11 and Figure 12, which are used as the input field for the coupled hygro-mechanical analysis. The field-dependent shear modulus is input as calculated by Equation (7).

**Figure 13:**
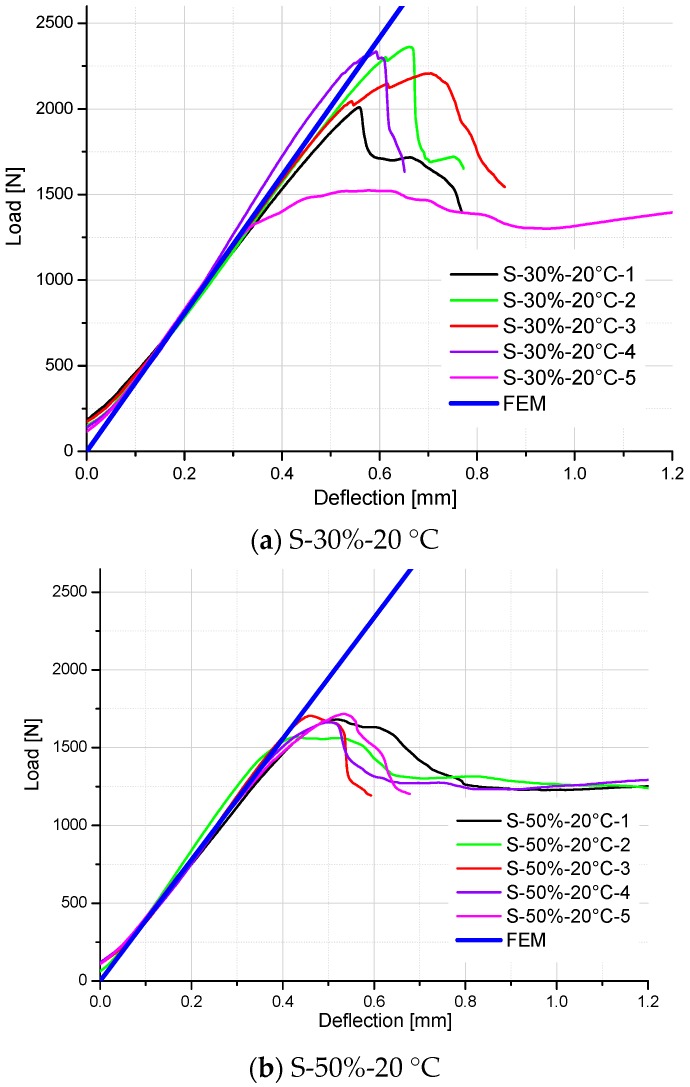
Comparison of load–deflection curves between FE analysis and test results of S-30%-20 °C specimens and S-50%-20 °C specimens.

Comparison between FE results and test data of S-30%-20 °C specimens and S-50%-20 °C specimens is shown in Figure 13a,b respectively. Good agreements on the slopes of load-displacement curves are achieved for these two groups of specimens, which prove that the predictive Equation (7) is relatively accurate to simulate the stiffness of GFRP specimens with other moisture contents.

For the GFRP specimens tested at 20 °C and during the moisture desorption process, the same inverse parameter identification method is employed to determine the environment-dependent interlaminar shear modulus of FRP laminates. The predictive equation is fitted and validated by the middle two exposure time intervals (unsaturated conditions 30% and 50%), as follows:

(8) $$G_{23}=G_{13}=-\frac{50\times M_{t}}{M_{\infty }}+850\;$$

For the FRP specimens tested at 40 °C and in the moisture absorption process, the predictive equation is as follows:

(9) $$G_{23}=G_{13}=-\frac{450\times M_{t}}{M_{\infty }}+1050\;$$

For the FRP specimens tested at 40 °C and in the moisture desorption process, the predictive equation is as follows:

(10) $$G_{23}=G_{13}=-\frac{250\times M_{t}}{M_{\infty }}+850\;$$

**Figure 14:**
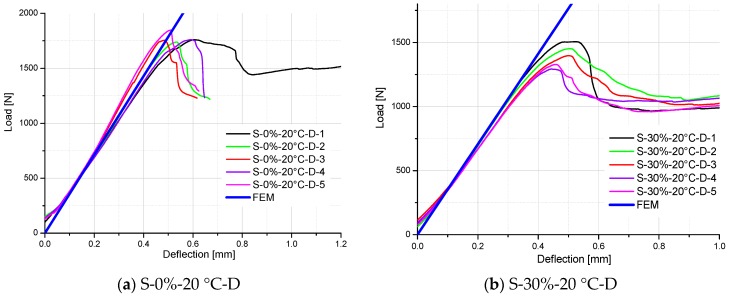

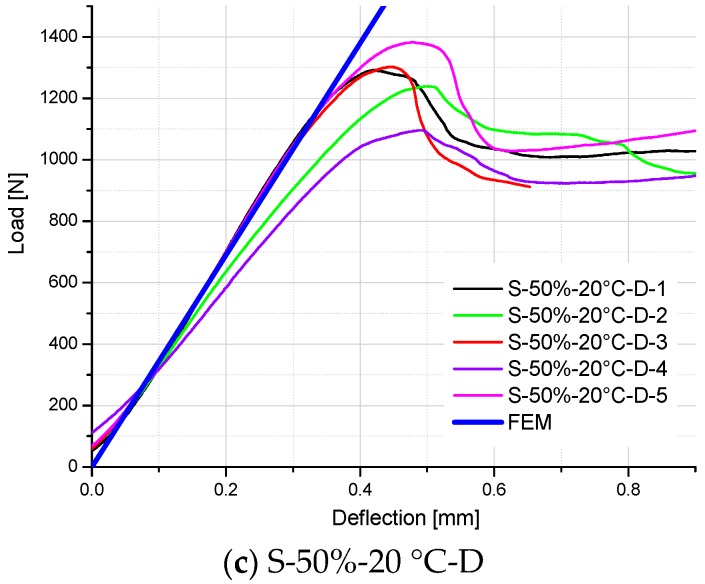
Comparison of load–deflection curves between FE analysis and test results of specimens tested at 20 °C and in the moisture desorption process.

**Figure 15:**
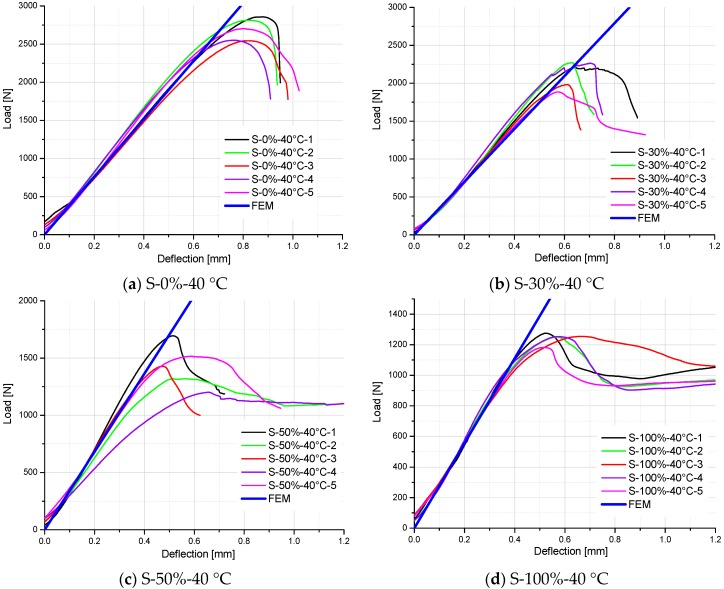
Comparison of load–deflection curves between FE analysis and test results of specimens tested at 40 °C and in the moisture absorption process.

**Figure 16:**
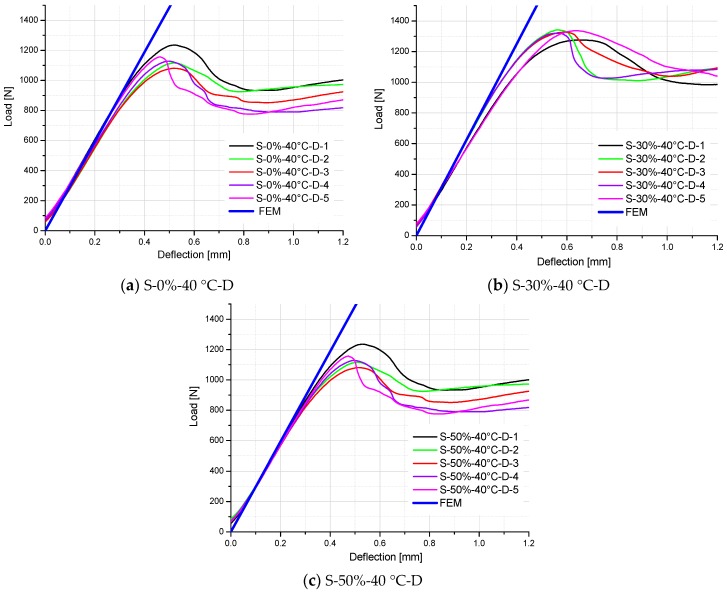
Comparison of load–deflection curves between FE analysis and test results of specimens tested at 40 °C and in the moisture desorption process.

For easy comparison, the stiffness of specimens is listed in Table 6, which are the slopes of load–deflection curves for the test and FE results. As shown in Table 5 and Figure 14, Figure 15 and Figure 16, a good agreement on the stiffness of specimens is evident between the FE predicted curves and test results. However, there are some exceptions (S-50%-20 °C-2, S-50%-20 °C-4, S-50%-40 °C-2, and S-50%-40 °C-4 specimens), which significantly deviate from other specimens in the same test group. It can be attributed to the non-homogeneity of GFRP laminate specimens, which influence the stiffness of the material. It also influences the moisture absorption property (different moisture content at the same aging time), and correspondingly degrades the material stiffness. Excluding these exceptions, predictive equations of moisture-dependent interlaminar shear modulus are acceptably reliable. Hence, they can be employed as input material properties of a hygro-mechanical FE model to analyze the environment-dependent mechanical behaviors of complex FRP components, joints, and structures in the future works.

**Figure 17:**
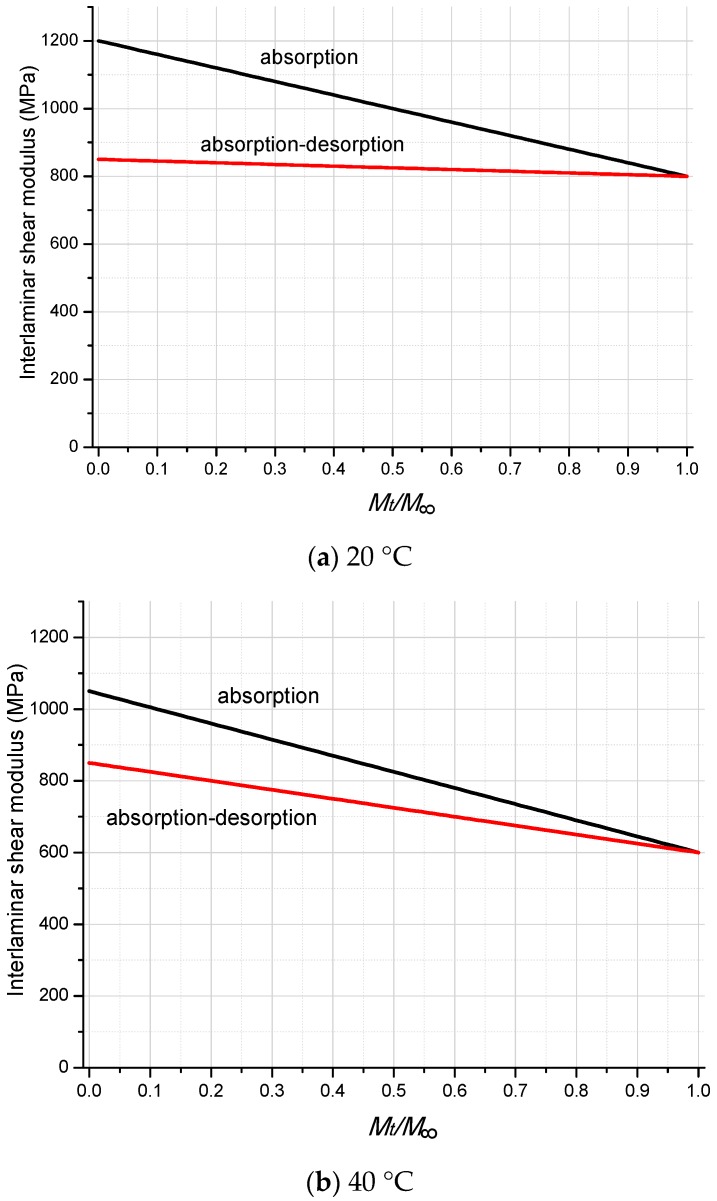
Degradation on the interlaminar shear modulus of GFRP laminates.

Figure 17 illustrates the degradation tendency of the interlaminar shear modulus of FRP laminates due to moisture diffusion and temperature. For the specimens tested at 20 °C, a dramatic drop on interlaminar shear modulus is found from the unconditioned dry specimen (1200 MPa) to the moisture-saturated specimen (800 MPa). After the moisture desorption process, a slight recovery is found for the shear modulus of S-0%-20 °C-D specimens (850 MPa). In total, a 29.2% decrease of interlaminar shear modulus is obtained for the specimens enduring one cycle of the moisture absorption–desorption process. For the specimens tested at 40 °C, a similar tendency of interlaminar shear modulus loss is obtained, with a 42.9% decrease from the unconditioned dry specimens (1050 MPa) to the saturated specimens (600 MPa), and a 19% decrease for the specimens enduring one cycle of the moisture absorption–desorption process.

## 5. Conclusions

This paper describes the investigations on the environment-dependent shear properties of GFRP laminates and the coupled hygro-mechanical FE method. Detailed conclusions can be drawn as follows:

1. The short-beam shear strength of the moisture-saturated specimens tested at 20 °C is 15 MPa, which is 53.1% lower than that of the unconditioned dry specimens (32 MPa). One cycle of the moisture absorption–desorption process degraded the shear strength of GFRP laminates by 34.4%. The elevated test temperature (40 °C) did not significantly degrade the shear strength of the GFRP laminates with different moisture contents.
2. Based on the coupled hygro-mechanical FE analysis method, an inverse parameter identification approach to short-beam shear tests was developed and employed to determine the environment-dependent interlaminar shear modulus of GFRP laminates. This method was proven to be effective to determine the interlaminar shear modulus of FRP materials.
3. Compared with the unconditioned dry specimen, the interlaminar shear modulus of the moisture-saturated specimen tested at 20 °C and 40 °C decreased by 33.3% and 42.9%, respectively. One moisture absorption–desorption process induced a 29.2% loss of interlaminar shear modulus for specimens tested at 20 °C, and a 19% loss for specimens tested at 40 °C.
4. Predictive equations for moisture-dependent shear strength and the modulus of GFRP laminates were obtained in this research. These predictive equations can be used as input parameters for a coupled hygro-mechanical FE model, and contribute to the design code of FRP structures as far as the long-term performance is concerned.

## Figures and Tables

**Table 1:** Glass fiber-reinforced polymer (GFRP) laminate properties of EQX1200 (supplied by the manufacturer).

Product Name	Total Weight (g/m^2^)	Weight Uniformity (g/m^2^)				
		Yarn Roving				Knit Yarn
		0° (Warp)	+45°	90° (Weft)	−45°	
EQX 1200	1193	283	300	300	300	10

**Table 2:** Mechanical properties of GFRP laminates (supplied by the manufacturer).

Property	Tensile (ISO 527-4)		Compression (ISO 8515)		Flexural (ISO 14.125)	
Mean Value	Warp	Weft	Warp	Weft	Warp	Weft
Strength	331 MPa	314 MPa	220 MPa	200 MPa	473 MPa	433 MPa
Modulus	18 GPa	17 GPa	14 GPa	14 GPa	13 GPa	11 GPa

**Table 3:** GFRP laminate specimens for short-beam shear tests.

Specimen Identification		Test	*M_t_*/*M_∞_*	Test Temperature	After Desorption	Number of Specimens
Set-1	S-0%-20 °C	shear	0	20 °C	no	5
	S-0%-40 °C	shear	0	40 °C	no	5
Set-2	S-30%-20 °C	shear	30%	20 °C	no	5
	S-30%-40 °C	shear	30%	40 °C	no	5
Set-3	S-50%-20 °C	shear	50%	20 °C	no	5
	S-50%-40 °C	shear	50%	40 °C	no	5
Set-4	S-100%-20 °C	shear	100%	20 °C	no	5
	S-100%-40 °C	shear	100%	40 °C	no	5
Set-5	S-50%-20 °C-D	shear	50%	20 °C	yes	5
	S-50%-40 °C-D	shear	50%	40 °C	yes	5
Set-6	S-30%-20 °C-D	shear	30%	20 °C	yes	5
	S-30%-40 °C-D	shear	30%	40 °C	yes	5
Set-7	S-0%-20 °C-D	shear	0	20 °C	yes	5
	S-0%-40 °C-D	shear	0	40 °C	yes	5

**Table 4:** Short-beam shear strength degradation of FRP laminates.

Specimen Identification	Shear Strength * (MPa)	Standard Deviation (MPa)
S-0%-20 °C	32	2.74
S-0%-40 °C	31	1.34
S-30%-20 °C	26	1.70
S-30%-40 °C	25	1.89
S-50%-20 °C	19	0.59
S-50%-40 °C	16	2.10
S-100%-20 °C	15	0.24
S-100%-40 °C	14	0.41
S-50%-20 °C-D	15	1.08
S-50%-40 °C-D	13	0.41
S-30%-20 °C-D	16	0.76
S-30%-40 °C-D	15	0.21
S-0%-20 °C-D	21	0.35
S-0%-40 °C-D	19	0.36

* mean value of five specimens.

**Table 5:** Mechanical properties of materials for the FE model.

Property	FRP Laminates
E_33_ (MPa)	11,000
*ν*_12_	0.33
*ν*_23_	0.3
*ν*_13_	0.18
G_12_ (MPa)	6986

**Table 6:** Stiffness of specimens based on load–deflection curves (Unit: N/mm). FEM: finite element method.

	Specimen	1	2	3	4	5	Mean Value	FEM	Error (100%)
Identification									
S-0%-20 °C		4582	4323	3973	4337	4534	4350	4286	1.47
S-30%-20 °C		3535	3692	3682	4318	3878	3821	4027	5.39
S-50%-20 °C		3659	3981	4062	3840	3684	3845	3896	1.32
S-100%-20 °C		3185	3163	3223	3556	2355	3096	3333	7.64
S-0%-20 °C-D		3295	3374	3696	3154	3919	3488	3571	2.39
S-30%-20 °C-D		3530	3426	3175	3178	3212	3304	3529	6.80
S-50%-20 °C-D		3632	2865	3405	2506	3517	3185	3448	8.26
S-0%-40 °C		3864	4162	3692	3919	4129	3953	3797	3.95
S-30%-40 °C		3938	4320	3824	4287	3753	4024	3488	8.36
S-50%-40 °C		4044	3069	3518	2286	3187	3221	3409	5.84
S-100%-40 °C		2897	2896	2588	3073	2694	2830	2778	1.82
S-0%-40 °C-D		2831	2661	2583	2653	2758	2697	2970	2.70
S-30%-40 °C-D		2664	2916	2902	2898	2587	2793	3125	8.29
S-50%-40 °C-D		2831	2661	2583	2653	2758	2697	2970	6.41
